# Bringing evidence to policy to achieve health-related MDGs for all: justification and design of the EPI-4 project in China, India, Indonesia, and Vietnam

**DOI:** 10.3402/gha.v6i0.19650

**Published:** 2013-03-13

**Authors:** Sarah Thomsen, Nawi Ng, Xu Biao, Göran Bondjers, Hari Kusnanto, Nguyen Tanh Liem, Dileep Mavalankar, Mats Målqvist, Vinod Diwan

**Affiliations:** 1 Department of Public Health Sciences (Global health/IHCAR), Karolinska Institutet, Solna, Sweden; 2 Department of Public Health and Clinical Medicine, Umeå Centre for Global Health Research, Umeå University, Umeå, Sweden; 3 Department of Epidemiology, School of Public Health, Fudan University, Shanghai, China; 4 Sahlgrenska Academy, Gothenburg University, Gothenburg, Sweden; 5 Department of Public Health, Faculty of Medicine, Gadjah Mada University, Yogyakarta, Indonesia; 6 National Pediatric Hospital, Hanoi, Vietnam; 7 Indian Institute of Public Health, Gandhinagar, Ahmedabad, India; 8 Department of Women's and Children's Health, International Maternal and Child Health, University Hospital, Uppsala University, Uppsala, Sweden

**Keywords:** equity, Millennium Development Goals, social determinants of health, evidence to policy, network, Asia

## Abstract

**Background:**

The Millennium Development Goals (MDGs) are monitored using national-level statistics, which have shown substantial improvements in many countries. These statistics may be misleading, however, and may divert resources from disadvantaged populations within the same countries that are showing progress. The purpose of this article is to set out the relevance and design of the “Evidence for Policy and Implementation project (EPI-4)”. EPI-4 aims to contribute to the reduction of inequities in the achievement of health-related MDGs in China, India, Indonesia and Vietnam through the promotion of research-informed policymaking.

**Methods:**

Using a framework provided by the Commission on the Social Determinants of Health (CSDH), we compare national-level MDG targets and results, as well as their social and structural determinants, in China, India, Indonesia and Vietnam.

**Results:**

To understand country-level MDG achievements it is useful to analyze their social and structural determinants. This analysis is not sufficient, however, to understand within-country inequities. Specialized analyses are required for this purpose, as is discussion and debate of the results with policymakers, which is the aim of the EPI-4 project.

**Conclusion:**

Reducing health inequities requires sophisticated analyses to identify disadvantaged populations within and between countries, and to determine evidence-based solutions that will make a difference. The EPI-4 project hopes to contribute to this goal.

The purpose of this article is to provide the relevance and design of the ‘Evidence for Policy and Implementation project (EPI-4)’, which aims to reduce inequities in achievement of the health-related Millennium Development Goals (MDGs) in China, India, Indonesia, and Vietnam through the promotion of informing research evidence with policy in these four countries.

## Background

According to the World Health Organization (WHO), while there is still much to achieve, there have been significant, positive changes in health-related MDGs on the global and country levels since 1990 (1). Under-five mortality has declined by 35%, maternal mortality has decreased by 2.3% per year, skilled birth attendance has increased from 55 to 65%, neonatal mortality has decreased by 28%, mortality due to tuberculosis has decreased by one-third, and the HIV epidemic has stabilized. However, these achievements are often occurring in an inequitable manner. In other words, while overall progress has been made, gaps in achievements between and within many countries have not decreased. The poorest and most disadvantaged communities are the least likely to have benefited from achievements. For example: poor, rural children are less likely to have received measles vaccination than their richer, urban counterparts (2).

Some regions are particularly affected by inequity in the achievement of MDGs. In Southern Asia, the wealthiest women are five times more likely than the poorest to have been attended to by a trained health care worker when giving birth (2). These health inequities have been most marked in the countries where economic growth has been particularly inequitable. For example, in India, where the annual per capita growth rate has hovered around 8% for the last decade, use of antenatal care services increased by 12% from 1996 to 2008, but only 0.1% among the poor. At the same time, 37% of the population is living in poverty (in some states, over 50% of the population) (3). The conclusion can only be that economic growth may be necessary, but not sufficient for improving the health of all. Governments must also be prepared to invest the benefits of economic growth in services that will actively promote reductions of health inequity, such as public health care and public education (4).

Unfortunately, the use of MDG targets has led to a focus on improving the *proportion* of people benefiting in a particular aspect of welfare – increased income, education, health, sanitation, housing, etc. – rather than on equitable distribution of health. In the implementation of MDGs, across these different areas, such targeting has often led to efforts being placed on improving the welfare of those most easily reached, otherwise referred to as ‘cherry-picking’ (5). Health interventions associated with MDGs 4, 5, and 6, for instance, are mainly applied through established health services to which only a minority of the population have easy access, usually the same fraction for all interventions (6). In effect, the goal-oriented approach of the millennium development approach has created an incentive for governments to implement utilitarian approaches to health as opposed to universalist ones, in the hopes of achieving ‘trickle-down’ effects (7). The result of this has been to create a greater disparity between those lifted ‘above the poverty line’ and those left behind (8). Therefore, while national MDG targets are approached in many countries, and average welfare increases, so does inequity (9).

## Social determinants of health

**Fig. 1:**
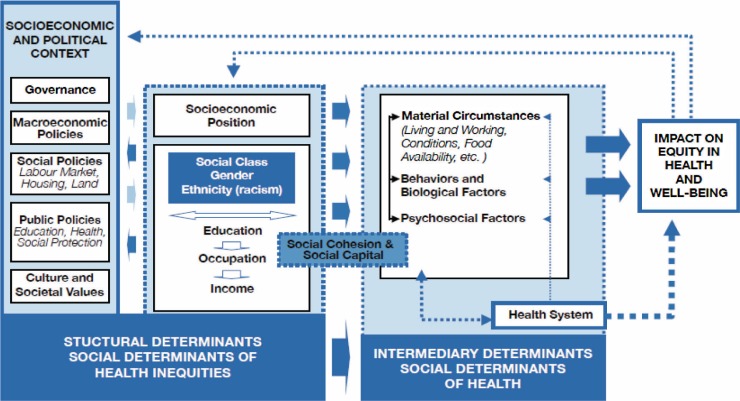
Social determinants of health framework (WHO, 2010). [Permission to reprint granted from WHO].

In 2009, the Sixty-second World Health Assembly passed resolution 64.14, which urged Member States to: ‘Tackle health inequities within and across countries through political commitment … to develop and implement goals and strategies to improve public health with a focus on health inequities, and to take into account health equity in all national policies that address social determinants of health (10).’ The Commission on the Social Determinants of Health (CSDH) has developed a framework for illustrating the mechanisms by which structural and social factors affect equity in health. This framework recognizes that there are multiple causes for health outcomes besides individual behavior and health service delivery (11). The model specifies three types of determinants of health: 1) the socioeconomic and political context, 2) structural determinants and socioeconomic position, and 3) intermediary determinants such as individual behavior and the health system (Fig. 1).

The socioeconomic and political context includes governance issues, such as corruption, policies around wealth transfer, education and health care, and cultural norms around men and women, and the acceptable behavior of children and adolescents. This environment generates or reinforces social stratification that defines individual socioeconomic position, including social class (often measured by proxy variables such as education, occupation and household income or wealth indices), gender, and ethnicity. Together, these make up the structural determinants of health. These structural determinants do not affect health directly. Rather, they are mediated by determinants such as material circumstance, individual behavior and biological factors, psychosocial factors, and the health system. Equity in health and well-being is proposed, in turn, to affect socioeconomic position and the socioeconomic and political context.

The majority of the world's poor (approximately 1.3 billion) now live in middle-income countries (MICs), such as China, Brazil, India, and Indonesia (12). This is a drastic change from 1990 when 93% of the world's poor were estimated to live in low-income countries (LICs). MICs today experience considerable inequity in the distribution of health services, and other specific health challenges, such as those caused by effects of rapid industrial and urban growth.

Below we compare national-level MDG targets and results for four emerging economies: China, India, Indonesia, and Vietnam. Together, these four countries represent about 42% of the world's population, with GDP growth rates of over 6% (13). At the same time, they have varied results in terms of social and health outcomes, including MDG targets. In order to illustrate the role of social determinants of health, we contrast these results with structural and intermediary determinants of health and health inequity for these same countries.

The latest MDG statistics from 2010 show reductions of 50% or more in under-five mortality and infant mortality across the four countries (Table 1). Immunization against measles is at nearly 100% in China and Vietnam. India and Indonesia have also made great strides (32 and 53% improvements, respectively). Similarly, maternal mortality rates have decreased by over 60% in each of these countries, although the overall rates are still unacceptably high in India (200/100,000) and Indonesia (220/100,000), in particular. When looking at some of the most important intermediary determinants of maternal mortality, it becomes clear why India and Indonesia are, on the whole, far from reaching their targets. India has an inadequate percentage of births attended by skilled health personnel (52.7%), low contraceptive use among married women (54.8%), high levels of unmet need (20.5%), and few women who have attended at least four antenatal care visits (26.9%), although the latter has improved by 51% in the last 20 years. Indonesia has better coverage of births by skilled personnel (79.4%) and contraceptive use (61.4%), but low antenatal care coverage (at least 4 visits=55.4%) and a very high adolescent birth rate (52.3%). There are other structural factors that also contribute to India and Indonesia's low performance in achieving MDGs that we will explore below.

**Table 1:** Percentage change from 1990 to 2010 for MDGs 4 and 5 in four Asian countries

	China			India			Indonesia			Vietnam		
				
Millennium Development Goals (MDGs)	1990	2010	% chg	1990	2010	% chg	1990	2010	% chg	1990	2010	% chg
Goal 4: Reduce child mortality												
Target 4.A: Reduce by two-thirds, between 1990 and 2015, the under-five mortality rate												
4.1. Under-five mortality rate per 1,000 live births	48	18	−63	115	63	−45	85	35	−59	51	23	−55
4.2. Infant mortality rate (0–1 year) per 1,000 live births	38	16	−58	81	48	−41	56	27	−52	37	19	−49
4.3. Children, 1-year-old, immunized against measles (%)	98	99	1	56	74	32	58	89	53	88	98	11
Goal 5: Improve maternal health												
Target 5.A: Reduce by three quarters, between 1990 and 2015, the maternal mortality ratio												
5.1. Maternal mortality ratio per 100,000 live births	120	37	−69	600	200	−67	600	220	−63	240	59	−75
5.2. Births attended by skilled health personnel (%)	94	99.3^12^	6	34.2^3^	52.7^10^	54	40.7	79.4^10^	95	77.1^5^	87.7^9^	14
Target 5.B: Achieve, by 2015, universal access to reproductive health												
5.3. Current contraceptive use among married women aged 15–49 years (%)	84.6^2^	84.6^9^	–	40.7^3^	54.8^11^	35	49.7^1^	61.4^10^	24	65^4^	77.8^13^	20
5.4. Adolescent birth rate per 1,000 women	16	6.2^12^	−61	76^1^	38.5	−49	66.2^2^	52.3^8^	−21	38^1^	35^12^	−8
5.5.a. Antenatal care coverage (at least one visit)	69.7^2^	92.2^12^	32	61.9^3^	75.2^11^	21	76.3^1^	93.3^10^	22	70.6^5^	90.8^9^	29
5.5.b. Antenatal care coverage (at least four visits)	NA	NA	NA	32^3^	26.9^11^	51.1	90^1^	55.4^10^	81.5	47^5^	15.2^7^	29.3
5.6 Unmet need for family planning (%)	3.3^2^	2.3^6^	−30	20.3^3^	20.5^11^	1	17^1^	13.1^10^	−23	8.4^5^	4.3^13^	−49

All data were downloaded from the Official United Nations site for MDG indicators (http://unstats.un.org/unsd/mdg/Data.aspx) and are available for years 1990 and 2010, except some of the data from the years

1 1991

2 1992

3 1993

4 1994

5 1997

6 2001

7 2002

8 2005

9 2006

10 2007

11 2008

12 2009

13 2011

The status of tuberculosis is still very tenuous in three of the four countries. While China has made significant advances on a national level (TB prevalence 108/100,000), India, Indonesia, and Vietnam still have TB prevalence rates of 256, 289, and 334/100,000, respectively. Although TB treatment success rates area around 90% in all countries (95% in China), the TB detection rate under DOTS is still worryingly low in India (59%), Indonesia (66%), and Vietnam (54%).

Table 2 also indicates another problem with country reporting, which is that there is little national data for some of the MDG indicators on HIV and malaria. In some cases, the country reports, such as the UNAIDS country progress reports (14), simply state that data on, for example, ‘sexual intercourse with more than one partner in the past 12 months’ are “irrelevant”.

**Table 2:** Percentage change from 1990 to 2010 for MDG 6 in four Asian countries

	China			India			Indonesia			Vietnam		
				
Millennium Development Goals (MDGs)	1990	2010	% chg	1990	2010	% chg	1990	2010	% chg	1990	2010	% chg
Goal 6: Combat HIV/AIDS, malaria, and other diseases												
Target 6.A: Have halted by 2015 and begun to reverse the spread of HIV/AIDS												
6.1.a. HIV prevalence among population aged 15–24 years (%)^a^	<0.1	0.1	>100	0.1	0.3	300	<0.1	0.2	>200	<0.1	0.4	>400
6.1.b. HIV incidence among population aged 15–24 years (%)^a^	<0.1	<0.1	Small	<0.1	<0.1	Small	<0.1	<0.1	Small	<0.1	<0.1	Small
6.2. Condom use during the last sexual intercourse among men and women aged 15–49 years who had more than one sexual partner^a^	NA	NA	NA	NA	67; 62^3^	NA	NA	NA	NA	NA	**92.9;**^6^ **N/A**	NA
6.3. Population aged 15–24 years with comprehensive correct knowledge of HIV/AIDS (% men;% women)^a^	NA	50; 55^4^	NA	NA	44; 35^6^	NA	NA	14; 15^4^	NA	NA	44; 41^6^	NA
6.4. Ratio of school attendance of orphans to school attendance of non-orphans aged 10–14 years^a^	NA	NA	NA	NA	0.72^3^	NA	NA	0.94^4^	NA	NA	NA	NA
Target 6.B: Achieve, by 2010, universal access to treatment for HIV/AIDS for all those who need it												
6.5. Antiretroviral therapy coverage among people with advanced HIV infection (%)^b^	NA	32	NA	NA	NA	NA	NA	24	NA	NA	52	NA
Target 6.C: Have halted by 2015 and begun to reverse the incidence of malaria and other major diseases												
6.6. Malaria death rate per 100,000 population, all ages^b^	NA	0^5^	NA	NA	2^5^	NA	NA	2^5^	NA	NA	0^5^	NA
6.7. Proportion of children under five sleeping under insecticide-treated bednets^c^	NA	NA	NA	NA	NA	NA	NA	3^4^	NA	NA	5^2^	NA
6.8. Proportion of children under five with fever who are treated with appropriate anti-malarial drugs^b^	NA	NA	NA	12^1^	8.2^3^		4.4^1^	0.8^4^		6.5^1^	2.6^3^	
6.9.a. Annual TB incidence rate/100,000 population^b^	153	78	−49	216	185	−14	189	189	0	204	199	−2
6.9.b. TB prevalence rate per 100,000 population^b^	215	108	−50	459	256	−44	423	289	−32	396	334	−16
6.9.c. TB death rate per year per 100,000 population^b^	19	4.1	−78	38	26	−32	51	27	−47	44	34	−23
6.10.a. TB detection rate under DOTS (%)^b^	21	87	314	80	59	−26	21	66	214	37	54	46
6.10.b. TB treatment success rate under DOTS (%)^b^		95^6^			88^6^			91^6^			92^6^	

The data were downloaded from

a UNAIDS data website (http://www.unaids.org/en/dataanalysis/knowyourepidemic/);

b the Official United Nations site for MDG indicators (http://unstats.un.org/unsd/mdg/Data.aspx);

c UNICEF (http://www.unicef.org/statistics/index_countrystats.html); and are available for year 1990 and 2010, except some of the data from years

1 2000

2 2005

3 2006

4 2007

5 2008

6 2009

Moving backwards through the CSDH model, we now turn to some selected ‘intermediary determinants’ of health (Table 3). Starting with the health system, China has dedicated twice as much of its GDP on health as Indonesia (5.1% vs. 2.6%), but less than Vietnam (6.8%). However, out-of-pocket expenditures (out of total expenditures on health) are reportedly similar in China (36.6%) and Indonesia (38.3%). In India and Vietnam, approximately 60% of all expenditures on health are out-of-pocket. Coverage of community health workers, the first line of primary health care, is below 1 per 1,000 in India (0.05), Indonesia (0.001), and China (0.83) (no information available for Vietnam). Interestingly, there are more physicians than nurses/midwives per 1,000 people in China (1.42 vs. 1.38) and Vietnam (1.22 vs. 1.0). In Indonesia (0.29 vs. 2.04) and India (0.65 vs. 1), the ratio is more geared toward the mid-level providers than physicians.

**Table 3:** Intermediary determinants of health in four Asian countries

Intermediary determinants	China	India	Indonesia	Vietnam
Material circumstances				
GNI* per capita in PPP* terms (constant 2005 international $)^a^	7476	3468	3716	2805
Unemployment, total (% of total labor force)^b^	4.3^6^	4.4^2^	7.1	2.4^5^
% Population using improved drinking water sources in 2010^c^	91	92	82	95
% Population using improved sanitation facilities in 2010^c^	64	34	54	76
Behavioral and biological factors				
Life expectancy at birth in year in 2011^a^	73.5	65.4	69.4	75.2
Smoking prevalence in 2009 (% men;% women)^b^	51.2; 2.3	26.3; 3.6	61.3; 5.1	48.2; 1.6
Exclusive breastfeeding (% of children under 6 months)^b^	27.6^5^	46.4^3^	15.3^4^	16.9^3^
Depth of hunger in 2008 (kilocalories per person per day)^b^	250	240	220	240
Prevalence of wasting (% of children under five)^b^	2.3	20^3^	14.8^4^	9.7^5^
Health system				
Health expenditure, total (% of GDP*) in 2010^b^	5.1	4.1	2.6	6.8
Out-of-pocket health expenditure (% of total exp. on health) in 2010^b^	36.6	61.2	38.3	57.6
Out-of-pocket health expenditure (% of private exp. on health) in 2010^b^	78.9	86.4	75.1	92.7
Community health workers (per 1,000 people)^b^	0.83^6^	0.05^2^	0.001^1^	NA
Nurses and midwives (per 1,000 people)^b^	1.38^6^	1.0^5^	2.04^4^	1.0^5^
Physicians (per 1,000 people)^b^	1.42^6^	0.65^6^	0.29^4^	1.22^5^

* GNI=Gross national income; PPP=Purchasing power parity; GDP=Gross national product. The data were downloaded from the

a International Human Development Indicators (http://hdr.undp.org);

b The World Bank (http://data.worldbank.org/topic/health); and

c Official United Nations site for MDG indicators (http://unstats.un.org/unsd/mdg/Data.aspx).

All data are from year 2010, except stated differently in the left column and some of the data are from years

1 2003

2 2005

3 2006

4 2007

5 2008

6 2009

Individual behavioral and biological factors, psychosocial factors and material circumstances also have an effect on equity in health, both directly and through use of health services. Smoking levels amongst men in Indonesia (61.3%), China (51.2%), and Vietnam (48.2%) are extremely high. Exclusive breastfeeding in the first 6 months of a child's life is relatively high in India (46.4%), compared to China (27.6%), Vietnam (16.9%), or Indonesia (15.3%). Use of ‘improved drinking sources’ is fairly high on a national level: 90% in China, India, and Vietnam, and 82% in Indonesia. However, ‘improved sanitation’ is poor in all countries, with large variations: 76% in Vietnam, 64% in China, 54% in Indonesia, and a very low 34% in India. The prevalence of wasting in children under five – an indicator of poor access to food – is very high in India (20%) and Indonesia (14.8%). In Vietnam almost one in ten children exhibit wasting; in China this figure is 2.3%.

The CSDH framework, which is based on hundreds of studies conducted over 20–30 years, posits that social position is the most important determinant of health inequity (11). The actual mechanism for this is not completely clear, but it is clear that education, ethnicity, social status, gender, income, and occupation are linked to many health outcomes. The link may be ‘social capital’, which is represented by the resources that an individual has access to through his or her social environment, which is determined by the above variables. Living in an urban slum environment is one indicator of socio-economic position. The proportion of the population living in urban populations in the four countries is highest in China (47.8%) and Indonesia (44.6%). India (30.3%) and Vietnam (31%) have relatively smaller urban populations (Table 4).^1^ On the other hand, Vietnam has the highest proportion of urban slum residents (35.2%). In China and India, urban slum populations are around 30% and it is 23% in Indonesia.

**Table 4:** Selected structural determinants related to socioeconomic position in four Asian countries

Structural determinants – socio-economic position	China	India	Indonesia	Vietnam
Population and demographic indicators				
Population in thousands in 2011^a^	1,347,565.3	1,241,492	242,325.6	88,792
% Population living in urban area in 2011^a^	47.8	30.3	44.6	31
% Slum population as% of urban population in 2009^b^	29.1	29.4	23	35.2
Occupation, income, and education indicators				
Poverty headcount ratio (% under national poverty line)^b^	2.8^1^	29.8	13.3	14.5^4^
Net enrollment ratio in primary education^b^	NA	98.2^4^	99.1	98.1
Adult literacy rate in men and women aged above 15^a^	94^5^	62.8^2^	92.2^4^	92.8^5^
Human Development Index in 2011^a^	0.687	0.547	0.617	0.593
Gender				
Gender Inequality Index in 2011^a^	0.209	0.617	0.505	0.305
Gender Parity Index in primary level enrollment^b^	1.03	1^4^	1.02	0.94
Population with at least secondary education, female/male ratio^a^	0.778	0.528	0.778	0.884
Ratio of female to male labor force participation rate in 2009	0.845	0.404	0.605	0.894
% of girls aged 15–19 who have had children or are currently pregnant^c^	NA	16^2^	9.5^3^	NA

The data were downloaded from the

a International Human Development Indicators (http://hdr.undp.org);

b Official United Nations site for MDG indicators (http://unstats.un.org/unsd/mdg/Data.aspx);

c World DataBank (http://databank.worldbank.org/data/home.aspx).

All data are from year 2010, except stated differently in the left column, and some of the data are from years

1 2004

2 2006

3 2007

4 2008

5 2009

The Human Development Index (HDI) is a composite measure of: 1) education, 2) standard of living, and 3) length and quality of life, with 1 being the highest level of human development according to these aspects. In 2011, China (0.687) was considered to have a ‘medium–high’ human development level, whereas Indonesia (0.617), Vietnam (0.593), and India (0.547) were all considered to have ‘low–medium’ human development levels. These ‘scores’ on the HDI are reflected in poverty and education statistics for the four countries. The poverty headcount ratio is measured as the proportion of the population that is living under the national poverty level. The highest proportion of poor is found in India (29.8%), followed by Indonesia (13.3%) and China (2.8%). Net enrollment in primary school is almost 100% in all countries (data not available in China), and adult literacy is over 90% except in India (62.8%).

Attitudes and norms regarding men's and women's roles and responsibilities in society are strongly related to health behaviors and outcomes (15). The expression of norms in a society can be measured in many different ways. The Gender Inequality Index reflects inequalities in achievement between men and women in reproductive health, educational attainment, and the labor market, with 0 indicating perfect equality. Gender inequality in these areas is worst in India (0.617) and Indonesia (0.505). China (0.209) and Vietnam (0.305) are much closer to reaching equality.^2^ This index may go a long way to explaining the differences in achievement in MDG 4 and 5 between the four countries. For example, labor participation for men and women is an important indicator of the ability of women to generate income, which is related to how household resources are spent (on health care costs for children or food, for example). The ratio of female to male labor participation is fairly high in China (0.845) and Vietnam (0.894), but low in Indonesia (0.605), and very low in India (0.404).

Education of girls is also a strong determinant of health outcomes for the whole family. Gender parity in primary school (enrolment of girls to boys) is at (or over) 1 in all countries but Vietnam (0.94), and the ratio of females to males with a secondary education is much lower in all four countries: 0.778 in China, 0.528 in India, 0.778 in Indonesia, and 0.884 in Vietnam, with 1 being perfect parity. This means, for example, that for every woman with a secondary school education in India there are two men with at least that level of education.

The reproductive health of women is partially determined by patterns of early marriage. The longer they wait to marry, the longer they tend to wait to begin childbearing, and the longer they can stay in school, thus increasing educational levels amongst women. The teenage pregnancy rates (aged 15–19) were 16% in India in 2006 and 9% in Indonesia in 2007 (data not available in China and Vietnam). Thus, gender norms that are reflected in low levels of women's achievement in secondary school, low participation in the labor market, high levels of teenage pregnancy, and the high levels of poverty in India and Indonesia are likely strong social determinants of the poor health results reported above.

In the CSDH framework, the socioeconomic and political structure of a country is purported to create the conditions that make possible differences based on socioeconomic position. For example, policies around education and social protection can create an enabling or disabling environment for different segments of the population to attend school, or for women to work. Thus, the high ratio of female to male participation in the labor market in Vietnam discussed above (89%) is likely related to the fact that the state subsidizes day care for children below school age, which is not the case in the other three Asian countries in this study, although all require employers to allow parental leave for at least 3 months (Table 5). Similarly, legislation often reflects cultural and social values that, as we have seen above, have an effect on behaviors and ultimately health. Thus, the high legal age at marriage in China, i.e. 20 years, is probably a protective factor against adolescent pregnancies, but also is an enabling factor for higher education among women seen in this country, along with the benefits this provides in health.

**Table 5:** Selected structural determinants related to the socioeconomic and political context in four Asian countries

Socioeconomic and political context	China	India	Indonesia	Vietnam
Governance				
Corruption Perception Index in 2011^a^	3.6	3.1	3.0	2.9
Macro-economic Policies				
Real GDP growth rate,%^b^	10.4	9.6	6.2	6.8
Public Policies				
Free public primary education^c^	yes	yes	yes	yes
Social Policies				
Minimum maternity leave in months^d^	3	3	3	4
State subsidization of childcare for children under school age^c^	no	no	no	yes
Cultural and social values				
Abortion legal^e^	yes	yes	Yes/limited	yes
Legal age at marriage for women^f^	20	18	16	18

a Transparency International (http://cpi.transparency.org/);

b The World Bank (http://data.worldbank.org/indicator/NY.GDP.MKTP.KD.ZG);

c Women, Business, and the Law (http://wbl.worldbank.org/data);

d ILO Conditions of Work and Employment (http://www.ilo.org/travail/lang–en/index.htm);

e Annual Review of Population Law (http://www.hsph.harvard.edu/population/annual_review.htm);

f Social Institutions and Gender Index (http://stats.oecd.org/Index.aspx?datasetcode=GIDDB2012).

All data are from year 2010, except when stated differently in the left column.

Other structural determinants that have been identified as important for health are governance, and macroeconomic policies. The Corruption Perception Index (CPI) has been used since 1995 to track perceptions of corruption in the public sector within countries. Since corruption is difficult to identify and trace, perceptions of corruption have been found to be more reliable. A score of 10 indicates no perceived corruption (most closely achieved in New Zealand, with 9.5). All four countries’ CPIs indicate low confidence in the public sector's ability to govern (all around 3), which may affect how worthwhile the average person thinks it is to be involved in the political processes in these countries. This, in turn, will affect the social standing of that person, or the group to which he/she belongs, according to the CSDH framework.

The final statistic that we present for the four countries is the annual growth rate of the gross domestic product (GDP). All have very high growth rates: 10.4% for China, 9.6% for India, 6.8% for Vietnam, and 6.2% for Indonesia in 2010. This indicates that there may be financial resources available to create the necessary social and structural conditions to improve the health and welfare of the populations of these Asian countries.

This review of selected indicators of social determinants of health in four Asian countries has allowed us to identify potential causes and determinants of ill-health. However, it is not sufficient to remain on this level. The differences *within* countries are often greater than the differences between them. Therefore, the use of national targets to reflect achievement of the MDGs is, as we discussed in the introduction, misleading at best and destructive at worst (5, 9). Subsequently, sub-national (provincial/state or district level) analyses to identify populations that are disadvantaged in relation to achievement of the MDGs and to disentangle the effects of different determinants on inequity in achievement of the MDGs are necessary. Finally, trend analyses are necessary to identify whether or not inequity gaps between different populations (i.e. the rich/poor, minority/majority, urban/rural) are increasing or decreasing, and whether the rate of these changes is greater or less in the different groups. This information will allow policymakers to pinpoint the greatest causes and determinants of inequity in achievement of the MDGs in their countries. Below we present the EPI-4 project, which is designed to help policymakers to do this in China, India, Indonesia, and Vietnam.

## The EPI-4 project

Increasing the use of research evidence in policymaking and implementation is widely recognized as a critical aspect to achieving health for all by 2015 (16). Doing so will require creating more effective mechanisms to bridge the know–do gap and address implementation issues (17). Facilitating factors to effectively link research to action are personal contacts between researchers and policy makers, timeliness and relevance of the research, and producing the research in a format that is actionable with clear policy recommendations and implications for implementation into practice (18, 19). Research syntheses should be context-specific and include evidence, modifying factors, needs, values, costs, and availability of resources. The research syntheses should address both the know–do gap and optimal ways of effective implementation (20).

The World Health Organization's Task Force on Research Priorities has called for more use of research in identifying and evaluating policy options to reduce health inequities (21). EPI-4 (Evidence for Policy and Implementation) was designed to increase capacity to make evidence-informed decisions on policies and implementation for health for disadvantaged groups in relation to MDGs 4, 5, and 6 in China, India, Indonesia, and Vietnam. The project will identify and use networks in each country to discuss evidence on inequity in achievement of the health-related MDGs and to plan for evidence-based interventions to reduce inequities. The evidence will be gathered and analyzed by researchers based at four Swedish universities: Karolinska Institutet, Gothenburg University, Umeå University, and Uppsala University, working in conjunction with longstanding partners in the four countries – Fudan University and Peking University, China; University of Gadjah Mada, Indonesia; the Public Health Institute of India; and the National Pediatric Hospital in Vietnam – and the ministries of health in these countries. The researchers will conduct systematic reviews to identify the most disadvantaged groups in relation to MDGs 4, 5, and 6 in each country (not all countries will look at each MDG outcome). We will also conduct secondary data analyses of large, existing datasets in each country, using the CSDH framework as a basis. These analyses will include: 1) descriptive statistics to show discrepancies in MDGs 4, 5, and 6 achievements across different population sub-groups within each country to fill gaps identified in the literature review; 2) regression analyses to identify the most important sources of inequity for various health outcomes, stratified by different groups; and 3) trend analyses of longitudinal or repeated cross-sectional data to determine whether or not equity has increased or decreased over time, and, if so, which groups have benefited. These results will be discussed in small network meetings of 10–15 persons consisting of academicians, policymakers, and other civil society representatives (such as non-governmental organizations) in each country. Results will be published in international, peer-reviewed, open-access journals, and results will also be summarized in research briefs. The project will end with a regional conference with high-level policymakers convened to discuss realistic approaches to reducing inequity in maternal and child health and infectious disease control and treatment.

## Footnotes

1 Differences in the definition of ‘urban’ may also be somewhat responsible for these differences.

2 As a comparison, the country that was closest to reaching gender equality according to this definition in 2011 received a score of 0.049 (Sweden).
